# Novel gut bacteria species *Paenibacillus ilasis* with phosphorus degrading and soluble starch hydrolysis abilities isolated from fresh feces of rhinoceros

**DOI:** 10.1038/s41598-025-06760-w

**Published:** 2025-07-01

**Authors:** Xue Li, Shuyu Zuo, Ming Li, Qin Li, Lei Su

**Affiliations:** 1 NHC Key Laboratory of Human Disease Comparative Medicine, National Human Diseases Animal Model Resource Center, International center for technology and innovation of animal model, Institute of Laboratory Animal Science, Chinese Academy of Medical Sciences (CAMS) and Peking Union Medical College (PUMC), Beijing, 100021 China; 2 College of Biological Sciences, China Agricultural University, Beijing, 100193 China; 3 Institute of Animal Science, Chinese Academy of Agricultural Sciences, Technology Support Platform, Beijing, 100193 China

**Keywords:** Organophosphorus solubilization, Soluble starch hydrolysis, Polyphasic taxonomy, *Paenibacillus ilasis* sp. nov., Microbiology, Systems biology

## Abstract

The genus *Paenibacillus*, known for its diverse sources, is a valuable reservoir of antimicrobial compounds, enzymes and other valuable chemicals, with applications in medicine, agriculture, and bioremediation. Despite this, *Paenibacillus* strains, particularly those isolated from unique environments, remain underexplored, limiting our understanding of their potential, capabilities and taxonomic classifications. The gut microbiome of large herbivores, such as rhinoceroses, harbors underexplored microbial diversity with unique metabolic capabilities. In this study, a Gram-stain-negative, facultatively aerobic, motile, spore-forming, rod-shaped bacterial strain, NGMCC 1.200843^T^ (= CGMCC 1.64763^T^ = JCM 37214^T^), was isolated from fresh rhinoceros feces and characterized its taxonomic status and metabolic potential. Phylogenetic, phenotypic, and chemotaxonomic analyses confirmed the isolate as a novel species within the genus *Paenibacillus*, closely related to *Paenibacillus lautus* DSM 3035^T^ (98.62% 16S rRNA gene similarity). The average nucleotide identity (ANI) and the digital DNA–DNA hybridization values were below the threshold for species delineation. The major cellular fatty acids were anteiso-C_15:0_, C_16:0_ and iso-C_16:0_ (> 10%) and the polar lipid profile contained diphosphatidylglycerol (DPG), phosphatidylglycerol (PG), phosphatidylethanolamine (PE), two unidentified phospholipids (PL1–2) and one phosphatidyl choline (PC). The total DNA G + C content was 49.69 mol%. The isolate exhibited significant phosphate solubilization and starch hydrolysis activities in plate assays, suggesting a role in nutrient cycling within the rhinoceros gut. We propose the name *Paenibacillus ilasis* sp. nov. for this strain. These findings enhance our understanding of gut microbial diversity in herbivores and lay the foundation for future applications in agriculture or industry.

**Supplementary Information:**

The online version contains supplementary material available at 10.1038/s41598-025-06760-w.

## Introduction

The genus *Paenibacillus*, initially classified within the genus *Bacillus* (first described in 1872 with *B. subtilis* as its type species), was formally established as a distinct taxon by Ash et al.^1^, with *Paenibacillus polymyxa* designated as the type species. This reclassification was primarily driven by phylogenetic analyses of 16S rRNA gene sequences and unique phenotypic characteristics. Members of the genus *Paenibacillus*, currently comprising over 324 validly published species (https://lpsn.dsmz.de/genus/Paenibacillus, up to May 27th, 2025)^2^, are characterized by Gram reaction diversity (mostly Gram-positive, with some species appearing Gram-negative or variable), the formation of elliptical endospores within swollen sporangium (predominantly positioned centrally or subterminally), DNA G + C content ranging from 39 to 59 mol%, facultatively anaerobic or strictly aerobic, 12-methyltetradecanoic acid (anteiso-C_15:0_) as the major cellular fatty acid, and menaquinone-7 (MK-7) as the principal isoprenoid quinone^3^. Strains of the genus *Paenibacillus* have been isolated from diverse environments^4^, including soil, water, plant rhizosphere, animals, and clinical samples. However, there are currently no literature reports of the isolation of *Paenibacillus* species from fresh rhinoceros feces, suggesting that microbial diversity in this particular niche remains underexplored. Species of this genus have been extensively studied for their functional diversity, such as promoting plant growth by dissolving phosphorus, fixing nitrogen, or producing plant hormones^5^. In addition, many strains produce antimicrobial compounds that have potential applications in medicine and agriculture^6^, or enzymes for bioremediation and industrial processes^7–9^. Despite this, *Paenibacillus* species from non-traditional sources, such as the gut of wild animals, remain understudied and may harbor new species with unique metabolic abilities.

The animal gut microbiota is a complex and dynamic ecosystem that is essential for the health and fundamental physiological processes of the host. These microbiota significantly impact animal health by breaking down dietary substrates to extract nutrients, promote host development, modulate the immune system, and protect the host from pathogens^10^. Especially in herbivores such as rhinos, the gut microbiota provides energy and nutrients to the host by breaking down plant matter rich in fiber and complex carbohydrates, producing short-chain fatty acids and other metabolites^11,12^. Recent advances in sequencing and culture-based omics have illuminated the diversity and ecological roles of rhinoceros gut microbiota. For instance, sequencing studies have revealed distinct microbial profiles between wild and captive black rhinoceroses, with low microbial diversity among captive individuals lacking fibro degrading bacteria common in wild rhinos^13^. Fecal microbiota studies of white rhinoceros have shown that *Firmicutes* and *Bacteroidetes* dominate fibrous fermentation^14^. Comparisons of gut microbiomes and metabolomes in captive rhinoceroses suggest a link to iron overload disease^15^. In addition, advances have been made in culture-based approaches, such as a cellulose-decomposing strain SS35 and a novel *Bacteroides* species from fresh rhinoceros feces^16,17^. These studies show that the combination of sequencing and culture methods can more comprehensively reveal the ecological function and health significance of rhino gut microbes and provide important data support for the protection of endangered species.

Compared with indepth studies on the human microbiota, wildlife microbiomes have received comparatively less attention in studies, with over 75% of their microbial composition remains uncharacterized^18^. This indicates that the wildlife microbiome is a huge treasure trove, with significant potential for discovering novel microbial taxa, genes, enzymes, antimicrobial agents, and probiotics. During surveyed intestinal microbial resources from animals as mice, alpaca, rhinoceros, marmot, etc., many new species of intestinal microorganisms have been discovered in our laboratory, including new species of fungi and bacteria^19–22^. In this study, we studied the microbial community in the intestines of rhinoceroses using culturomics and successfully isolated a novel bacterium capable of P solubilization and starch hydrolysis. The aim of this study was to elucidate the taxonomic position of strain NGMCC 1.200843^T^ based on detailed polyphasic studies. This research will provide novel potential strain with significant application potential and economic value in the fields of agriculture, food, and biotechnology.

## Results

### Phenotypic and physiological characterization

**Fig. 1:**
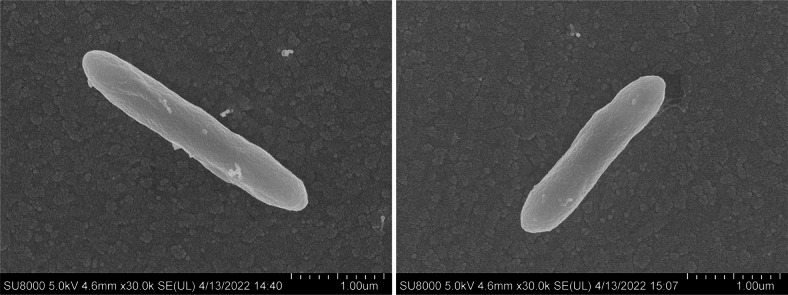
Scanning electron micrograph of strain NGMCC 1.200843^T^. Bar = 1.0 μm.

Strain NGMCC 1.200843^T^ was isolated from fresh rhinoceros feces and cultured aerobically on mGAM medium. Colonies appeared milky-white with low convex shapes, glossy surfaces, slightly irregular edges, and diameters of 1.0–2.0 mm after 24 h of incubation. Cells were facultative aerobe, sporulating under acidic stress (pH 6.0), Gram-negative, motile rods (0.4–0.5 μm × 2.5–4.0 μm, Fig. 1). Growth occurred at 20–37 °C (optimum 30 °C), pH 6.0–8.0 (optimum pH 7.0), and 0–2% NaCl concentrations (w/v; optimum 1%). The isolate tests positive for catalase, oxidase, and nitrate reduction to nitrite. Table 1 highlights the key features distinguishing strain NGMCC 1.200843^T^ from closely related *Paenibacillus* species. A comprehensive comparison of phenotypic, chemotaxonomic, and genomic features between strain NGMCC 1.200843^T^ and related *Paenibacillus* species is provided in Supplementary Table S1. The strain NGMCC 1.200843^T^ can metabolize a range of substrates, including sugars such as d-arabinose, l-xylose, adonitol, d-melezitose, d-lyxose, l/d-fucose, d-tagatose, l-arabitol, rhamnose, and melibiose, as well as non-sugar substrates like alkaline phosphatase, acid phosphatases, α-glucosidase, α-fucosidase, glycerol, 5-keto-d-gluconate, ONPG, and amygdalin. API ZYM assays detected alkaline phosphatase, acid phosphatase, and naphthol-AS-BI-phosphohydrolase activities, implicating active phosphate metabolism, and the strain also tested positive for starch hydrolysis. Distinctive features differentiating strain NGMCC 1.200843^T^ from four *Paenibacillus* type strains included its utilization of l-xylose, d-melezitose, d-lyxose, l/d-fucose, d-tagatose, l-arabitol, and 5-keto-d-gluconate. These metabolic differences, along with its Gram-negative staining and unique chemotaxonomic profile (Table 1), support its classification as a novel species within the genus.

**Table 1:** Key differentiating characteristics of strain NGMCC 1.200843^T^ and related type strains of the genus *Paenibacillus*.

Characteristics	1	2	3	4	5
Optimum pH	7.0	7.0	7.0	7.0	7.0
pH range	6.0–8.0	6.0–10.8	6.5–11.0	6.0–11.0	5.0–12.0
Temperature range (°C)	20–37	4–50	15–37	10–37	10–40
Optimum temperature (°C)	30	30	30	37	30
NaCl range (% w/v)	0–2	0–10	0–9	0–3	0–5
Optimum NaCl (%)	1	ND	0	1	0
Motility	+	ND	+	+	+
Endospore formation	+	ND	−	+	+
Cell shape	Rod	Rod	Rod	Rod	Rod
Cell length (μm)	2.5–4.0	1.6–8.0	1–1.2	4.0	1.6–3.3
Cell width (μm)	0.4–0.5	0.8–1.3	0.6–0.7	1.0	0.4–0.7
Gram staining	Negative	Positive	Positive	Negative	Positive
API ZYM results					
Alkaline phosphatase	+	ND	ND	−	+
Lipoidase (C14)	−	−	w	−	−
Acid phosphatases	+	ND	ND	−	−
α-Glucosidase	+	−	+	−	−
α-Fucosidase	+	ND	ND	−	−
API 50CH results					
Glycerol	+	+	+	+	−
Erythritol	+	−	−	+	−
d-arabinose	+	−	−	+	−
l-xylose	+	−	−	−	−
Adonitol	+	−	−	+	−
d-melezitose	+	−	−	−	−
d-lyxose	+	−	−	−	−
l-fucose	+	−	−	−	−
d-fucose	+	−	−	−	−
d-tagatose	+	−	−	−	−
l-arabitol	+	−	−	−	−
5-keto-d-gluconate	+	−	−	−	−
API 20E results					
ONPG hydrolysis	+	+	−	ND	+
Rhamnose	+	−	−	+	−
Melibiose	+	+	+	+	−
Amygdalin	+	+	+	+	w
Arabinose	+	−	+	+	ND

Strains: (1) NGMCC 1.200843^T^; (2) *Paenibacillus lautus* DSM 3035^T^; (3) *Paenibacillus glucanolyticus* DSM 5162^T^; (4) *Paenibacillus qingshengii* JCM 30613^T^; (5) *Paenibacillus solani* FJAT-22460^T^. Data were obtained in this study unless indicated. + positive, − negative, *w* weakly positive, *v* variable, *ND* no data available.

While most *Paenibacillus* species exhibit Gram-positive or Gram-variable, the Gram-negative profile of strain NGMCC 1.200843^T^ aligns with validated Gram-negative members of the genus, including *Paenibacillus lactis* DSM 15596^T^^23^, *Paenibacillus mobilis* KCTC 33848^T^^24^, *Paenibacillus qingshengii* JCM 30613^T^^25^. The Gram-negative cell wall structure of strain NGMCC 1.200843^T^ may reflect evolutionary adaptation to its gastrointestinal niche in rhinoceroses. These distinguishing physiological traits provide a foundation for genomic investigations into niche specialization and functional divergence within *Paenibacillus*.

### Chemotaxonomic characteristics

**Fig. 2:**
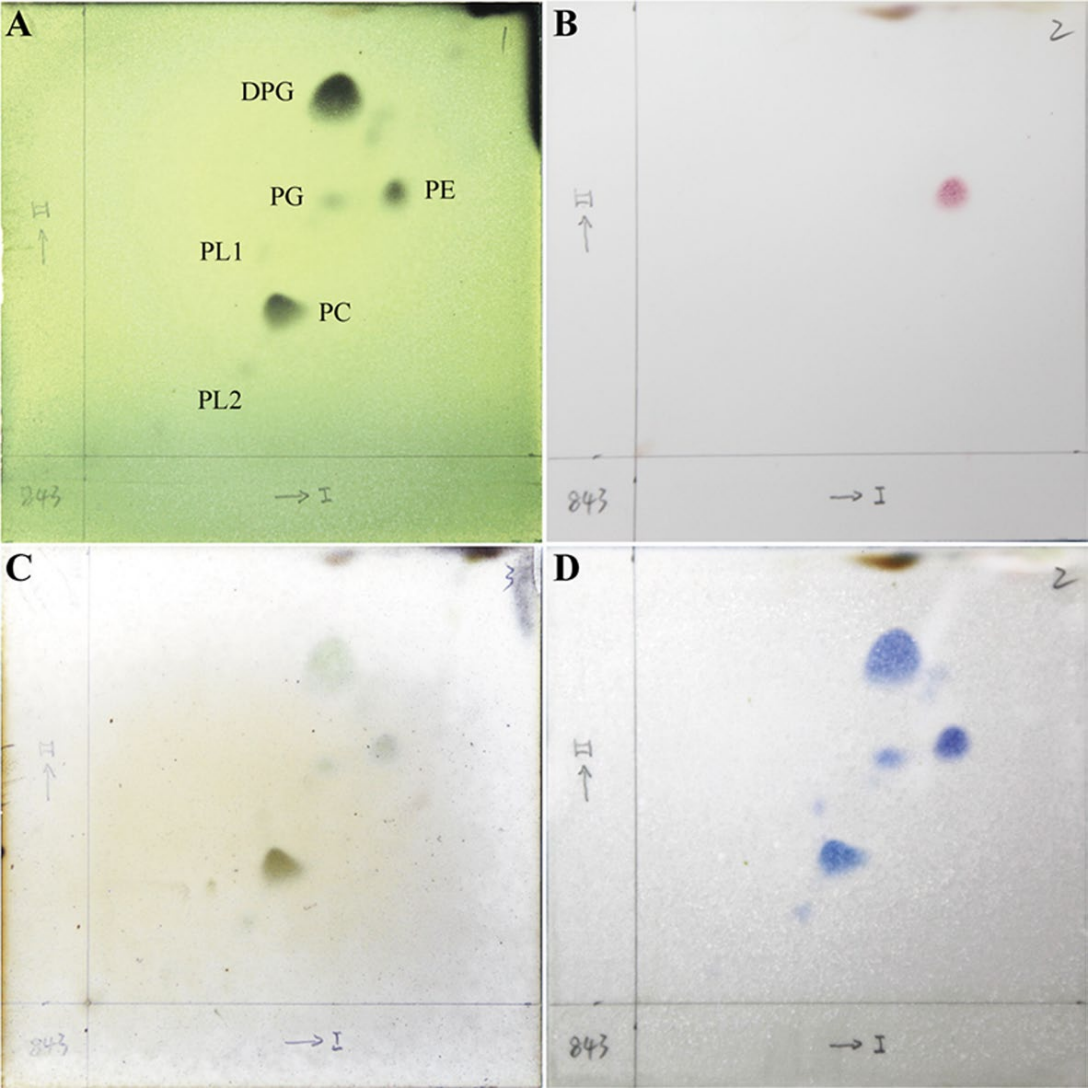
Polar lipids profile of strain NGMCC 1.200843^T^ after two-dimensional TLC and detection with (**A**) molybdophosphoric aicd, (**B**) ninhydrin, (**C**) α-naphthol and (**D**) molybdenum blue. *DPG* diphosphatidylglycerol, *PG* phosphatidylglycerol, *PE* phosphatidylethanolamine, *PL1-2* unidentified phospholipids, *PC* phosphatidyl choline.

The cellular fatty acid profile of strain NGMCC 1.200843^T^, characterized by saturated branched and straight-chain fatty acids (Table 2), consists of anteiso-C_15:0_ (33.39%), C_16:0_ (23.56%), iso-C_16:0_ (10.69%), iso-C_15:0_ (7.36%), and anteiso-C_17:0_ (6.54%). This profile aligns with those of other *Paenibacillus* species, with the predominance of anteiso-C_15:0_ being a typical feature of the genus *Paenibacillus*^26^. The primary polar lipids identified in strain NGMCC 1.200843^T^ include diphosphatidylglycerol (DPG), phosphatidylglycerol (PG), phosphatidylethanolamine (PE), two unidentified phospholipids (PL1 and PL2) and one phosphatidyl choline (PC) (Fig. 2). Strain NGMCC 1.200843^T^ shared core polar lipids (DPG, PG, PE, and PL1-2) with *Paenibacillus glucanolyticus* DSM 5162^T^^25^, *P. lautus* DSM 3035^T^^27^, *P. qingshengii* JCM 30613^T^^23^, *Paenibacillus solani* FJAT-22460^T^^28^. Notably, PC served as a distinguishing chemotaxonomic marker, as this lipid was absent in all closely related type strains, which provides critical evidence for its taxonomic distinction within the genus *Paenibacillus*. While DPG and PG are conserved polar lipids among *Paenibacillus* species, the presence of PC has rarely been reported in this genus. These chemotaxonomic findings supported the classification of strain NGMCC 1.200843^T^ within the genus *Paenibacillus.*

**Table 2:** Cellular fatty acid compositions of strains NGMCC 1.200843^T^ and other related *Paenibacillus* type strains.

Fatty acid	1	2	3	4	5
Saturated straight-chain					
C_14:0_	1.96	1.7	ND	ND	2.1
C_15:0_	ND	ND	ND	ND	ND
C_16:0_	23.56	19.1	2.3	5.7	12.1
C_18:0_	4.86	ND	ND	ND	1.0
Unsaturated straight-chain					
C_16:1_ ω11c	ND	4.2	2.3	1.2	1.3
C_16:1_ ω7c	ND	1.5	1.6	ND	ND
Saturated branched-chain					
Iso-C_14:0_	2.45	1.6	2.9	2.0	3.4
Iso-C_15:0_	7.36	2.6	4.7	3.3	5.3
Iso-C_16:0_	10.69	10.3	8.5	11.9	10.7
Iso-C_17:0_	4.78	2.3	ND	1.0	2.3
Anteiso-C_15:0_	33.39	47.3	69.4	65.1	51.4
Anteiso-C_17:0_	6.54	12.3	4.8	8.0	6.9

Strains: 1, NGMCC 1.200843^T^; 2, *P. lautus* DSM 3035^T^; 3, *P. glucanolyticus* DSM 5162^T^; 4, *P. qingshengii* JCM 30613^T^; 5, *P. solani* FJAT-22460^T^. Values ≥ 1% are shown; *ND* no data available.

### Phylogenetic analyses

**Fig. 3:**
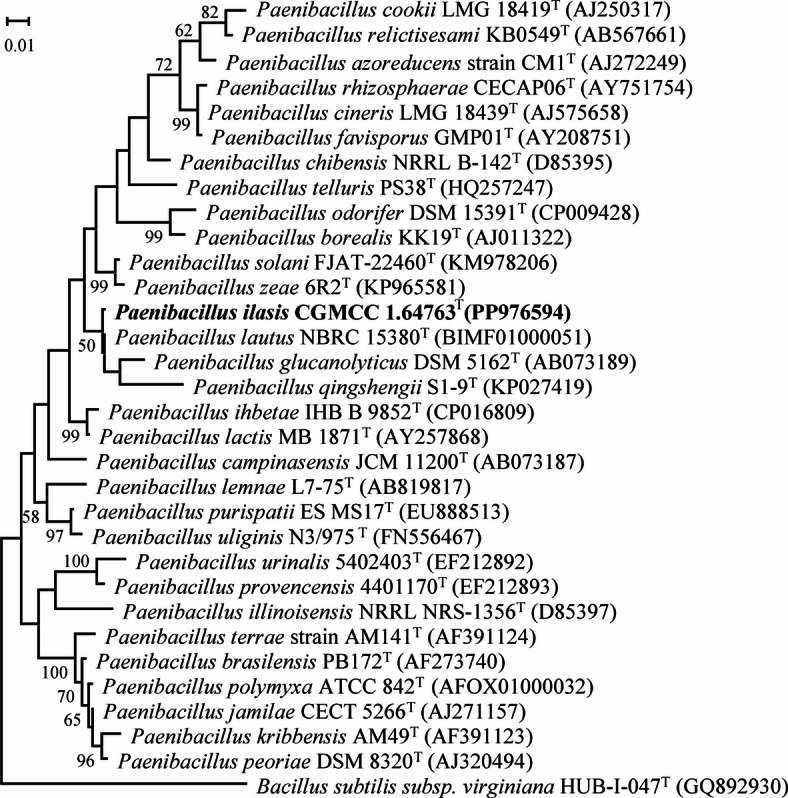
Maximum likelihood (ML) phylogenetic tree of strains NGMCC 1.200843^T^ with other type strains within *Paenibacillus* genus. *Bacillus subtilis* subsp. *Virginiana* HUB-1-047^T^ (GQ892930) is used as the outgroup. Numbers at nodes (bootstrap values) indicate percentages of 1000 replications and are shown where > 50%. Bar, 0.1 substitutions per nucleotide position.

**Fig. 4:**
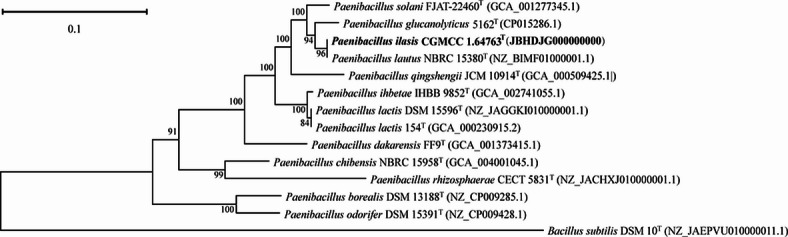
Genome phylogenomic tree showing the position of strain NGMCC 1.200843^T^. Bootstrap values are indicated at branch points based on 1000 iterations. *Bacillus subtilis* DSM 10^T^ (NZ_JAEPVU010000011.1) was used as an outgroup. Bar, 0.1 substitutions per nucleotide position.

The 16S rRNA gene sequence (1468 bp) of strain NGMCC 1.200843^T^ was obtained and analyzed. A BLAST search revealed low similarity with recognized *Paenibacillus* species. Strain NGMCC 1.200843^T^ showed the highest 16S rRNA gene sequence similarity to *P. lautus* DSM 3035^T^ (98.62%), followed by *P. glucanolyticus* DSM 5162^T^ (97.33%), *P. lactis* MB 1871^T^ (97.02%), and *P. solani* FJAT-22460^T^ (97.01%). The phylogenetic tree based on 16S rRNA gene sequences (Fig. 3) indicated that strain NGMCC 1.200843^T^ belongs to the genus *Paenibacillus,* forming a distinct phylogenetic lineage that diverges from closely related species, including *P. lautus* DSM 3035^T^, *P. glucanolyticus* DSM 5162^T^, and *P. qingshengii* JCM 30613^T^ with a bootstrap value of 50%. The homologous distance between strain NGMCC 1.200843^T^ and the standard strain *P. lautus* DSM 3035^T^, suggesting that strain NGMCC 1.200843^T^ was preliminarily identified as a *Paenibacillus*-like species. Additionally, the phylogenomic tree using 120 conserved single-copy marker proteins (GTDB framework) further resolved its taxonomic placement, with *Bacillus subtilis* DSM 10^T^ as an outgroup (Fig. 4). Further phylogenomic analysis confirmed that strain NGMCC 1.200843^T^ was affiliated with the genus *Paenibacillus*, showing the closest relation to *P. lautus* DSM 3035^T^. In summary, both 16S rRNA and phylogenomic topologies congruently identified *P. lautus* DSM 3035^T^ as its nearest neighbor, confirming genus-level affiliation while underscoring divergence sufficient for species demarcation.

### Identification of P-solubilizing ability

**Fig. 5:**
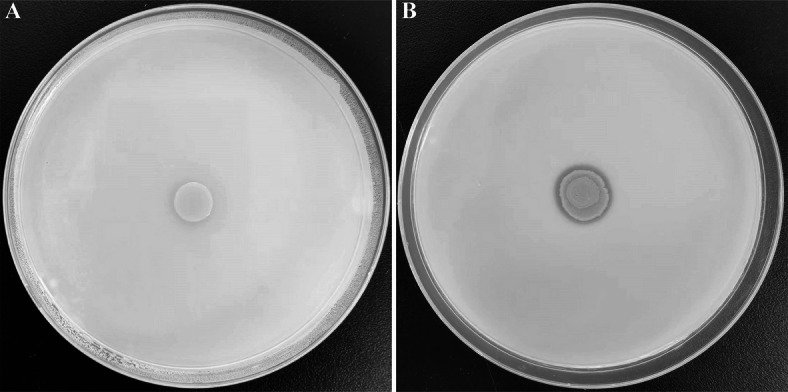
Qualitative analysis of organic P solubilization ability. (**A**) *Escherichia coli* L-7 as negative control; (**B**) Strain NGMCC 1.200843^T^ cultivated on lecithin as P source medium plates.

The phosphate-solubilizing capacity of strain NGMCC 1.200843^T^ was evaluated using a solid plate assay. On media containing organic phosphorus, the strain exhibited a colony diameter of 5.00 ± 0.34 mm and a clear halo zone surrounding the colony with a diameter of 29.00 ± 1.9 mm, resulting in a high phosphate solubilization index of 4.50 ± 0.30 (Table 3; Fig. 5). The finding indicates a significant ability to solubilize organic phosphorus. In contrast, no clear solubilization halos were observed when strain NGMCC 1.200843^T^ was incubated on media containing inorganic phosphorus (Fig. S1). The negative control, *Escherichia coli* L-7, did not produce a clear zone on organic phosphorus media.

**Table 3:** Qualitative analysis of bacteria strain NGMCC 1.200843^T^ phosphate release capacity.

Bacterial strains	Colony diameter (mm)	Halo zone diameter (zone of solubilization in mm)	Phosphate solubilization index (PSI)
NGMCC 1.200843	5.29 ± 0.34	29.04 ± 1.93	4.46 ± 0.30
Escherichia coli L-7	5.02 ± 0.18	5.16 ± 0.22	0.02 ± 0.01

### Identification of starch hydrolysis ability

**Fig. 6:**
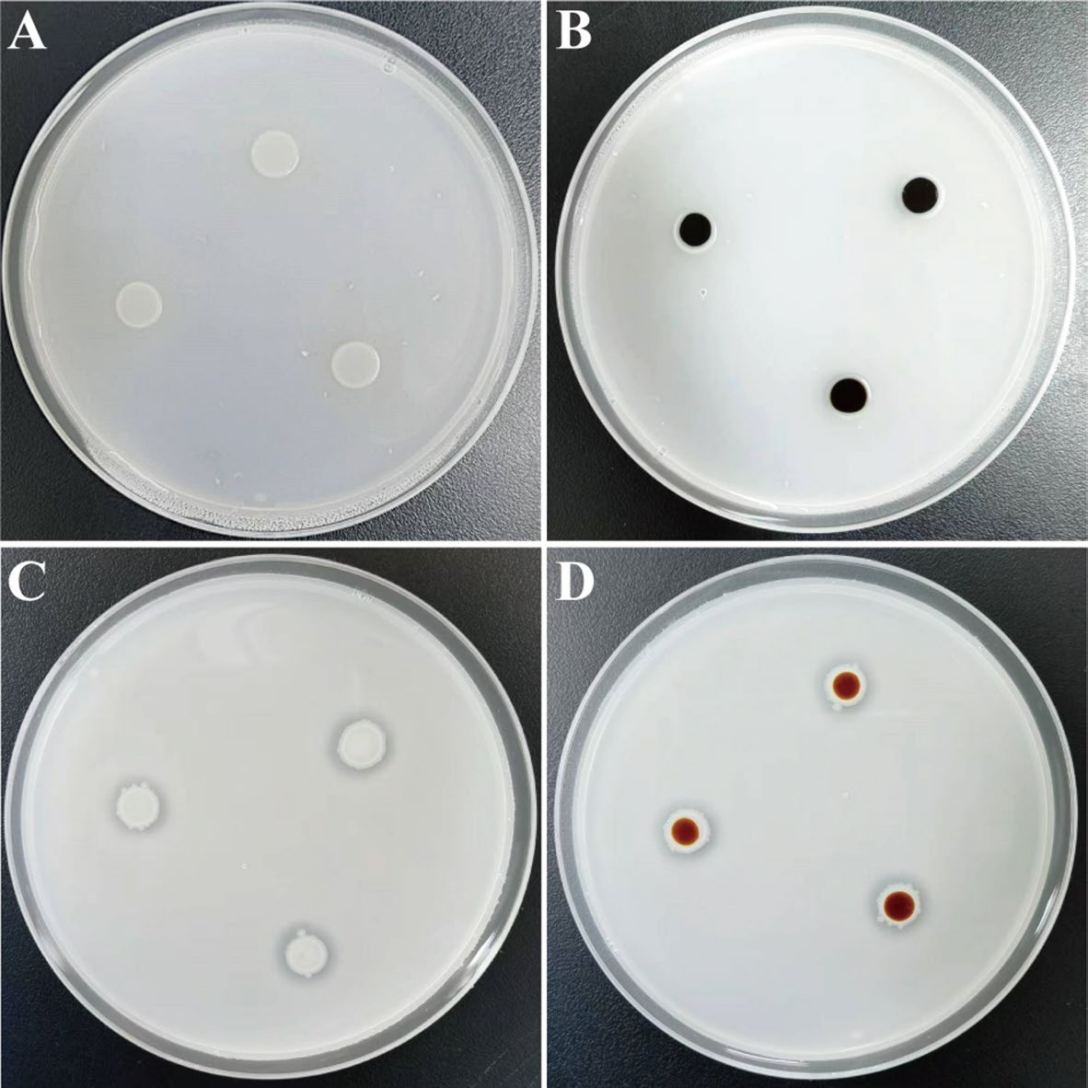
Qualitative analysis of starch hydrolysis ability by iodine staining on starch agar plates. The presence of a clear zone around culture growth indicates positive starch hydrolysis. (**A**,**B**) *Escherichia coli* L-7 as negative control, starch-hydrolytic activity before and after iodine application. (**C**,**D**) the positive starch hydrolytic activity of NGMCC 1.200843^T^ before and after addition of iodine solution.

Starch hydrolysis was assessed by culturing strain NGMCC 1.200843^T^ on a basal medium containing 1% soluble starch (Fig. 6). A prominent clear zone was observed surrounding the colonies, indicating effective starch degradation. Upon treatment with iodine solution, the colonies transitioned from milky white to red brown, confirming the hydrolysis of amylopectin within the soluble starch. In contrast, the control group consisting of *Escherichia coli* L-7 colonies displayed no clear zone, and the colonies turned black after iodine treatment, indicating the absence of starch hydrolysis. The above results demonstrate strain NGMCC 1.200843^T^ possesses the ability to hydrolyze soluble starch and secrete relevant extracellular enzymes.

### Genome analyses

**Fig. 7:**
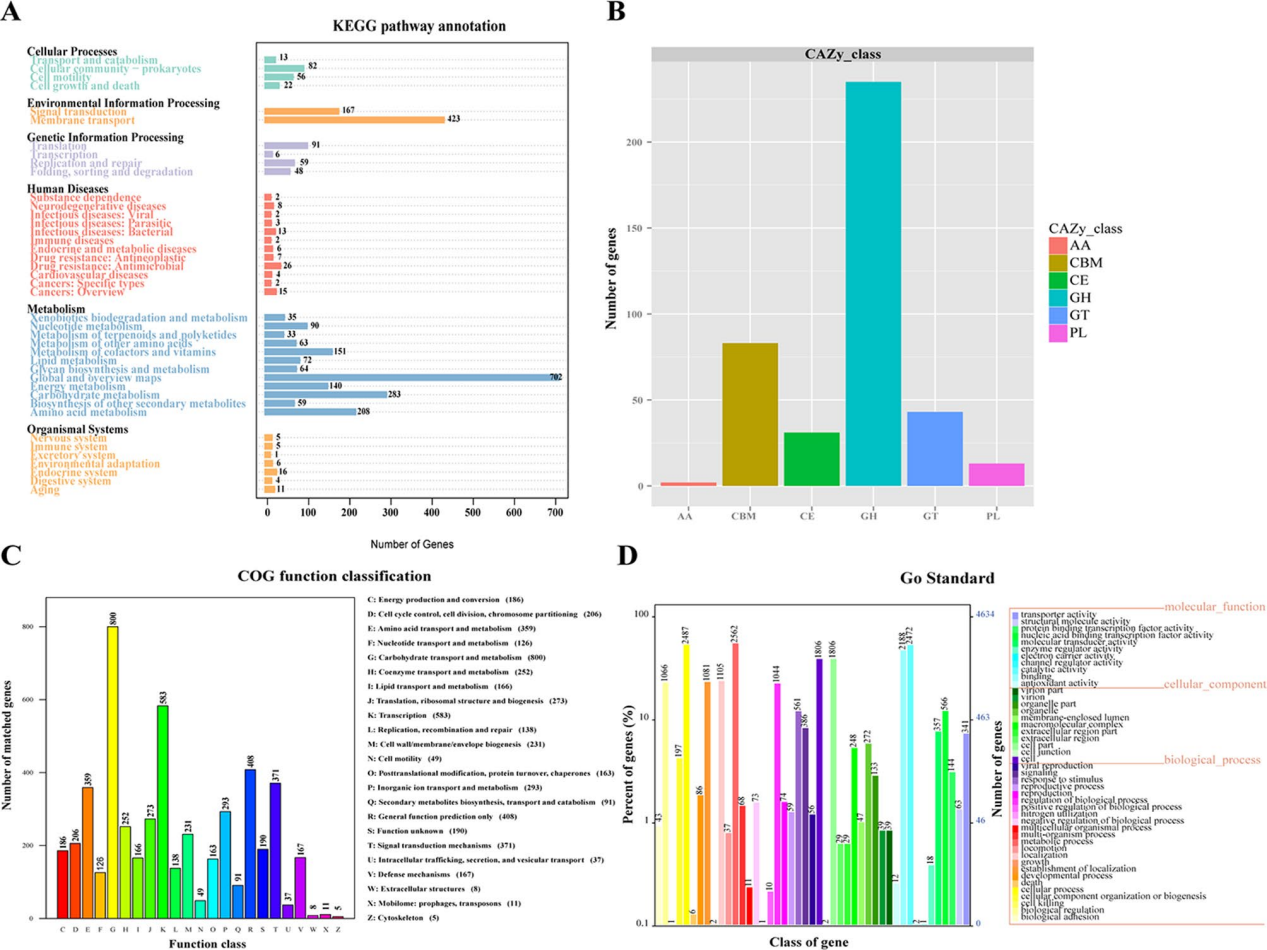
Genomic analysis of stain NGMCC 1.200843^T^. (**A**) KEGG function classification; (**B**) CAZy function classification; (**C**) COG function classification; (**D**) GO function classification.

The genome of strain NGMCC 1.200843^T^ comprises 14 contigs, totaling 7,228,608 bp in size, with G + C content of 49.69% and Scaffold N50 of 4,453,751 bp. CheckM v1.2.3 confirmed high genome quality with a completeness of 99.85%, contamination of 0.51%, and strain heterogeneity of 0%. The genome contains 9 rRNA genes (7 5S rRNA and 1 16S rRNA, with 1 23S rRNA gene) and 70 tRNA genes. A total of 6681 genes were predicted, with a cumulative coding sequence length of 6,288,009 bp, representing 86.99% of the total genes. The DNA G + C content of 49.69% falls within the range reported for the genus *Paenibacillus*^26^. The digital DNA–DNA hybridization (dDDH) values between strain NGMCC 1.200843^T^ and closely related type strains were *P. lautus* DSM 3035^T^ (68.50%), *P. glucanolyticus* DSM 5162^T^ (24.80%), *P. qingshengii* JCM 30613^T^ (21.8%) and *P. solani* FJAT-22460^T^ (27.50%) below the 70% species delineation threshold^29^ (Table 4). While the dDDH value with *P. lautus* DSM 3035^T^ (68.5%) approached the critical threshold, it remains insufficient for conspecific designation. The average nucleotide identity (ANI) analysis further resolved taxonomic boundaries. Strain NGMCC 1.200843^T^ exhibited an ANI value of 95.76% with *P. lautus* DSM 3035^T^, which lies within the “grey zone” (95–96%) for species delineation^30^. While this value slightly exceeds the widely accepted 95% threshold for bacterial species demarcation^31^, it remains below the stricter 96% cutoff established for highly conserved genera^32^. These genetic criteria should always be accompanied by a discriminant phenotypic property^33^. Notably, strain NGMCC 1.200843^T^ exhibits distinct phenotypic traits atypical for *Paenibacillus*: Gram-negative cell walls, unique polar lipid profile (presence of phosphatidyl choline) and Distinct metabolic capabilities (Utilization of niche carbon sources, including l-xylose, d-melezitose, d-lyxose, l-fucose, d-fucose, d-tagatose, l-arabitol, 5-keto-d-gluconate) further support its status as a novel species. These phenotypic divergences, combined with genomic data (dDDH, ANI, and phylogenomic analysis), unequivocally support the proposal of strain NGMCC 1.200843^T^. Gene Ontology (GO) analysis has three ontologies describing biological processes, cellular components, and molecular functions, helping to elucidate the biological functions behind genes. In GO annotation (Fig. 7), genes linked to biological processes constituted the largest category, followed by molecular function and cellular component. According to COG analysis, the 5113 predicated genes could be divided into 23 functional categories, with carbohydrate transport and metabolism representing the largest proportion. Carbohydrate-active enzymes (CAZymes) are a significant enzymes group that catalyze the degradation, modification, and conversion of carbohydrates, particularly in enhancing the efficiency of hydrolyzing biomass in bioenergy crops. Comparisons with the CAZy database revealed that strain NGMCC 1.200843^T^ contains 31 carbohydrate esterases, 235 glycoside hydrolases, 43 glycosyltransferases, 13 polysaccharide lyases, and 2 auxiliary activities.

**Table 4:** Average nucleotide identity and levels of DNA–DNA hybridization among the strain NGMCC 1.200843^T^ and related strains.

Query genome	Reference genome	ANI (%)	dDDH (%)
NGMCC 1.200843	*P. lautus* DSM 3035^T^ (NZ_BIMF01000001.1)	95.76	68.50
NGMCC 1.200843	*P. glucanolyticus* DSM 5162^T^ (CP015286.1)	81.61	24.80
NGMCC 1.200843	*P. qingshengii* JCM 10914^T^ (GCA_000509425.1)	77.05	21.10
NGMCC 1.200843	*P. solani* FJAT-22460^T^ (GCA_001277345.1)	83.52	27.50

### Gene function annotation

To explore the biotechnological potential of strain NGMCC 1.200843^T^, functional screening was performed from the genomic perspective. Based on KEGG annotation, genes related to phosphate solubilizing and starch hydrolysis were identified within the genome of strain NGMCC 1.200843^T^ as shown in Table S2. Generally, bacteria can solubilize insoluble P into the soluble form by producing organic acids and phosphatase, thereby enhancing P uptake in plants. Strain NGMCC 1.200843^T^ is capable of solubilizing organic P through several phosphatases, including pyrophosphatase, alkaline phosphatase and members of the haloacid dehalogenases (HAD) family. Starch breakdown by gut bacteria occurs through the combined action of amylases, which include α-amylase (EC 3.2.1.1) that hydrolyzes α-1,4-glycosidic bonds in starch; glucoamylase (EC 3.2.1.2), which acts on the non-reducing ends of amylose and amylopectin; and pullulanase (EC 3.2.1.3), responsible for cleaving the α-1,6-glycosidic bonds in amylopectin, along with amylopullulanases (APU; EC 3.2.1.41). In strain NGMCC 1.200843^T^, we identified the *amy* encoding the α-amylase.

Phosphonates (Pn) are organo-P molecules characterized by the highly stable C–P bond. The genes for phosphonate uptake and degradation were clustered in a 12.6 kb operon of seventeen genes, in alphabetical order, designated *phnA* to *phnQ*^34^. Strain NGMCC 1.200843^T^ carries part *phn* genes (*phn ABWX*) that are involved in the solubilization of organic phosphate. The *Phn CDEM* was not present, which may reflect the gene gain and loss events during the evolutionary process.

The *pst* (phosphate-specific transport) system is a key phosphate (Pi) transport system (ABC transporter system). Strain NGMCC 1.200843^T^ carry the *pst* operon (*pstSCAB*) along with the PhoP-PhoR signaling system. Within this system, *pstS* functions as a binding protein, while *pstC* and *pstA* are integral inner membrane proteins. The *PstB* is ATP binding protein, enabling the transport of extracellular substrates. The PhoP-PhoR, a two-component signal transduction system, regulates the expression of phosphonate uptake and C-P lyase activity in response to the phosphate deficiency.

### Description of ***Paenibacillus ilasis*** NGMCC 1.200843^T^

*Paenibacillus ilasis* (‘*ilasis*’ is. L. gen. n. ilas, referring to the discoverer’s unit abbreviation of type strain NGMCC 1.200843^T^, Institute of Laboratory Animal Sciences).

Cells were facultatively aerobic, Gram-negative rods with dimensions of 0.4–0.5 µm width × 2.5–4.0 µm length. Colonies on mGAM agar were milky white, glossy, low convex, and circular, measuring 1.0–2.0 mm in diameter after 24 h incubation at 30 °C. Spore formation was observed under acidic stress (pH 6.0), and it is motile. Growth occurs at 20–37 °C (optimum 30 °C), pH 6.0–8.0 (optimum pH 7.0), NaCl 0–2% (optimum 1.0%, w/v). Nitrate was reduced to nitrite. Catalase and oxidase reactions were positive, and urease reactions were negative. Gelatin, starch, ONPG, esculin ferric citrate can be hydrolyzed. Carbon sources include rhamnose, arabinose, d-lyxose, l-fucose, d-fucose, d-tagatose, d-arabitol, l-arabitol, glucose, mannose, *N*-acetylglucosamine, d-cellobiose, d-maltose, d-lactose, d-melibiose, d-sucrose, d-trehalose, d-melezitose, d-raffinose, glycogen, xylitol, gentiobiose, d-turanose, galactose, fructose, sorbose, dulcitol, d-arabinose, l-arabinose, ribose, d-xylose, l-xylose, adonitol, methyl-β-d-xylopyranoside, amygdalin, gluconate, 5-keto-d-gluconate, methyl-α-d-mannopyranoside, methyl-α-d-glucopyranoside, amygdalin, arbutin, salicin, inulin, mannitol, sorbitol, inositol, glycerol. Except for Lipoidase(C14), α-mannosidase, α-fucosidase, the cells are positive for alkaline phosphatase, esterase (C4), Lipid esterase (C8), leucine arylamidase, cystine arylamidase, chymotrypsin, acid phosphatases, naphthol-AS˗BI-phosphohydrolase, α-galactosidase, β-galactosidase, α-glucosidase, β-glucosidase, *N*-Acety-β-glucosaminidase. The major fatty acids are anteiso-C_15:0_, C_16:0_ and iso-C_16:0_ (> 10%). The predominant polar lipids are DPG, PG, PE, PL1-2 and PC 1.

The type strain is *Paenibacillus ilasis* NGMCC 1.200843^T^ (= JCM 37214^T^ = CGMCC 1.64763^T^), isolated from fresh feces of rhinoceros. The DNA G + C content of type strain is 49.69%. The 16S rRNA gene sequence and genome accession number are PP976594 and JBHDJG000000000, respectively.

## Discussion

In this study, *P. ilasis* NGMCC 1.200843^T^ was isolated from the fresh feces of male rhinoceros, representing the first documented *Paenibacillus* species originating from this ecologically distinct niche. Phenotypic and phylogenetic analyses unequivocally classified *P. ilasis* NGMCC 1.200843^T^ as a novel species within *Paenibacillus*. Its atypical phenotypic profile (most notably the Gram-negative reaction) challenges the traditional characterization of *Paenibacillus* as a strictly Gram-positive or Gram-variable genus. This observation aligns with emerging reports of validated Gram-negative members such as *P. qingshengii* JCM 30613^T^ and *P. mobilis* KCTC 33848^T^, suggesting that cell wall architecture in this genus exhibits previously underestimated diversity. Functional characterization through plate assays and genomic sequencing revealed its dual proficiency in phosphate solubilization and starch hydrolysis. These capabilities are highly relevant to the gut environment, as they contribute to the breakdown of complex dietary components. The degradation of lecithin, a phospholipid prevalent in plant and animal tissues, provides phosphorus essential for microbial metabolism, while starch hydrolysis releases energy-rich sugars that benefit both the microbiota and the host. The rhinoceros gut, as an underexplored reservoir of microbial diversity, embodies a complex interplay between host physiology and ecosystem nutrient cycling. As large herbivores, rhinoceroses consume vast quantities of plant biomass, predominantly composed of substrates recalcitrant to host enzymatic digestion, including recalcitrant polysaccharides and phytate-bound phosphorus-substrates. Within this environment, gut symbiotic microbes such as *P. ilasis* NGMCC 1.200843^T^ act as dynamic bioreactors, dismantling these polymers into bioavailable nutrients, thereby bridging host metabolism with broader ecological processes.

Phosphorus (P) is a vital macronutrient essential for energy metabolism, cellular function, and structural integrity across biological systems. In herbivorous animals like the rhinoceros, efficient utilization of dietary phosphorus, which is often locked in organic forms from plant material, is critical for nutrition and health, as it is involved in bone formation and energy metabolism, and this process relies heavily on microbial activity within the gut. *Paenibacillus ilasis* NGMCC 1.200843^T^ was isolated from rhinoceros feces and showed significant phosphorus-solubility and may play an important role in the rhino intestinal niche. *Paenibacillus ilasis* NGMCC 1.200843^T^ was confirmed to have alkaline and acidic phosphatase activity in API ZYM assays. On solid medium containing organic phosphorus, a transparent halo zone was formed around the colony with a phosphorus solubility index of 4.50 ± 0.30, indicating highly efficient phosphatase activity. This phenomenon may be attributed to several mechanisms, including the production of organic acids that lower the pH and facilitate phosphorus solubilization, the secretion of polysaccharides that enhance microbial adhesion and nutrient availability, or the activity of phosphatase enzymes that hydrolyze organic phosphorus compounds^35^. Genomic analysis further revealed genes associated with phosphorus lysis, including several phosphatases such as pyrophosphatase, alkaline phosphatase, members of haloacid dehalogenases (HAD) family, and the *phn-pst* system—a hybrid pathway for organic phosphate mineralization (*phnABWX*) and high-affinity inorganic phosphate transport (*pstSCAB*) (Tabe S2). These genetic adaptations enable *P. ilasis* NGMCC 1.200843^T^ to thrive in the nutrient-rich, competitive gut environment, where efficient phosphorus processing is essential. Herbivores, such as rhinos, need to convert organic phosphorus from food into inorganic phosphorus during digestion in order to be absorbed by the body. This process relies heavily on the role of the gut microbe, which secretes enzymes such as phosphatases to help break down organophosphate compounds. The inorganic phosphorus released after decomposition can be absorbed through the intestines and enter the bloodstream, where it can be used for bone growth, energy metabolism and other functions. The phosphorus solubilization ability of the *Paenibacillus* genus is primarily achieved through the secretion of organic acids (such as citric acid and oxalic acid) and phosphatase activity. Many species that have been separated from this genus have been confirmed to possess P-solubilizing capabilities, including *P. mucilaginosus*^36^, *P. elgii*^37^, *P. panacihumi*^38^, *P. kribbensis*^39^, *P. polymyxa*^40^, *P. macerans*^41^, and *P. xylanilyticus*^42^, and several unclassified strains. While the primary significance of *P. ilasis* NGMCC 1.200843^T^ lies in its ecological role within the rhino gut, this characteristic also underscores its potential as a beneficial agent for improving phosphorus availability in agricultural systems.

The gastrointestinal tract of herbivores harbors a rich repertoire of carbohydrate-active enzymes (CAZymes) that degrade complex plant polysaccharides into bioavailable nutrients, sustaining both microbial communities and their hosts. In this study, *P. ilasis* NGMCC 1.200843^T^ exhibited positive starch hydrolysis result in API 50CH physiological experiments, which was further confirmed by a culture plate color reaction experiment using 1% starch. The initial dextrin formed during this process is erythrodextrin, which produces a color progression from blue to violet to red brown upon iodine addition, confirming the presence of hydrolyzed starch products. Genomic and functional analyses further identified the *amy* gene encoding α-amylase. Within the animal gastrointestinal tract, starch, a major dietary polysaccharide, serves as a critical carbon source for the resident microbiota. This microbial dependency on starch hydrolysis is facilitated by enzymes such as α-amylases, which cleave α-1,4-glycosidic bonds to release glucose subunits for energy metabolism^43^. Previous research had underscored the starch hydrolysis capabilities of amylases within the genus *Paenibacillus*. For example, Tsusaki et al.^44^ successfully purified α-glucosidase (AGL) and α-amylase (AMY) from the culture supernatant of *Paenibacillus* sp. PP710, investigating their concerted reactions mechanism. Ikram et al.^45^ focused on optimizing cultural conditions for α-amylase production by *P. amylolyticus,* presenting this strain as a promising new source for enzyme production. Similarly, Rajesh et al.^46^ reported the identification of an α-amylase-encoding gene from a genomic DNA library of *Paenibacillus* sp., along with analysis of functional properties of the resulting protein. In this study, characteristic hydrolyzed starch of *P. ilasis* NGMCC 1.200843^T^, combined with the identification of the corresponding *amy* gene, positions it as a candidate for further exploration in industrial applications.

## Materials and methods

### Feces sampling and cultivable bacteria isolation

Strain NGMCC 1.200843^T^ was isolated from fresh feces of male rhinoceros in Beijing Zoo, Beijing, China. Fecal samples were processed in an anaerobic glove box (Shanghai Longyue Co., Ltd) with an atmosphere of 90% N_2_, 5% H_2_ and 5% CO_2_. Approximately 1 g of fecal sample was added to a sterile centrifuge tube containing 10 ml of sterile anaerobic phosphate-buffered saline (PBS) (pH 7.0, with 1% cysteine) (CORNING, USA). The mixture was shaken and filtered through cell sieves (70 μm and 40 µm) to facilitate bacterial detachment from the fecal residue. The bacterial suspension was treated following method of Li et al.^17^ and then spread onto yeast extract casein hydrolysate fatty acid (YCFA) medium^47^ and Modified Gifu Anaerobic Medium (MGAM; HB8518, Hopebio) agar plates, each supplemented with 5% sterile sheep blood and 10% rumen fluid. The plates were incubated at 37 °C for 15 days. The strain was stored both on mGAM slants at 4 °C and as suspensions in mGAM with 30% (v/v) glycerol at − 80 °C.

### Morphological and physiological characterization

Cell morphology was examined using a light microscope (BX53, Olympus) and scanning electron microscopy (SEM) (Merlin compact, ZEISSI). To assess sporulation, strain NGMCC 1.200843^T^ was cultured in mGAM medium at a pH of 6.0 under acidic stress conditions at 30 °C for 24 h. After incubation, samples were examined microscopically for spore formation. Growth conditions were tested in aerobic, anaerobic, and microaerophilic environments created using a bio˗incubator, AnaeroPack™˗Anaero, and MicroAero™˗MicroAero systems (Mitsubishi Gas Chemical Co, Inc). Growth was evaluated at varying temperatures (4 °C, 10 °C, 20 °C, 25 °C, 30 °C, 37 °C, 45 °C, 50 °C), pH values (ranging from 3.0 to 11.0 in increments of 1.0 pH units), and NaCl concentrations (from 0 to 8% at 1% increments). Gram reaction was determined with the bioMérieux Gram Stain kit (G1060, Solarbio). Catalase activity was assessed by observing bubble formation in 3% (v/v) hydrogen peroxide solution. Oxidase activity was tested using the 21st test of API 20NE. Carbon substrate utilization, acid production enzyme activities, and other physiological tests were conducted using API 50CH, API 20E and API ZYM (bioMérieux), following the instructions of the manufacturer.

### Chemotaxonomic characterization

Fatty acid methyl esters were analyzed using gas chromatography/mass spectrometry (Agilent Technologies 7890A GC System) in accordance with the Sherlock Microbial Identification System (MIDI)^48^ and the Sherlock 6.0 Software. Biomass for cellular acid analysis was obtained from a 48 h culture grown on mGAM plate at 37 °C. Polar lipids were extracted and identified using two-dimensional TLC, following the procedures established by Minnikin et al.^49^. The sample powder was separated in a methanol: chloroform solution, and the lower organic phase was concentrated for further use. The sample points on TLC plate, dried and stained with chromogenic agents such as molybdatophosphoric acid, ninhydrin, molybdenum blue, and α-naphthol. By comparing the colorimetric results and migration rates under different chromogenic agents with the standard chromatogram of polar lipids, the predominant polar lipid type of the sample was identified.

### 16S rRNA sequencing and phylogenetic analysis

The 16S rRNA gene of strain NGMCC 1.200843^T^ was amplified using universal bacterial primers 27F and 1492R^50^. The PCR products were subsequently purified and sequenced by BGI Genomics Co., Ltd. The resulting 16S rRNA gene sequence was compared with available sequences in GenBank database using the BLAST program (http://www.ncbi.nlm.nih.gov/BLAST/) to ascertain its approximate phylogenetic affiliation. The 16S rRNA gene sequence similarity threshold of 98.65% was applied as a preliminary criterion for species delineation, in accordance with recommendations for taxa requiring polyphasic taxonomic analysis^30^. Strains exhibiting less than 98.65% similarity were considered putative novel species candidates, requiring further validation through whole-genome comparisons (ANI/dDDH) and phenotypic characterization^33^. Multiple alignments of amino acid sequences for concatenated analyses were performed using Clustal_X software v2.0^51^. Sequence-based evolutionary distances were calculated with Kimura’s two-parameter model^52^. The phylogenetic tree was constructed using three algorithms: neighbor-joining (NJ)^53^, maximum-likelihood (ML)^54^, and maximum˗parsimony (MP)^55^, all implemented in MEGA_X software v10.2.6^56^. Bootstrap analysis with 1000 replications was conducted to assess the confidence levels for branches^57^. A phylogenomic tree was reconstructed using the bac120 marker set, following the methodology of Li et al.^17^ Bacterial marker genes (n = 120) for phylogenetic inference were identified with GTDB-Tk v0.3.3^58^. Genome sequences of *Paenibacillus* species, including *P. lautus* DSM 3035^T^ (NZ_BIMF01000001.1), *P. glucanolyticus* DSM 5162^T^ (CP015286.1), *P. qingshengii* JCM 30613^T^ (GCA_000509425.1), *P. solani* FJAT-22460^T^ (GCA_001277345.1), were retrieved from the NCBI database (https://www.ncbi.nlm.nih.gov/)^59^.

### Genome analyzes

The genomic DNA of strain NGMCC 1.200843^T^ was extracted from pure cultures using the TIANamp Bacteria DNA Kit (DP302, Tiangen), following the manufacturer’s instructions. The genome sequencing was performed on an Illumina NovaSeq PE150 platform at Novogene Co., Ltd. (Beijing, China) with an average sequencing depth of 65× coverage. The draft genomes was assembled using SOAPdenovo v2.04 (https://soap.genomics.org.cn/)^60^, SPAdes^61^, and ABySS^62^, then integrated with CISA software^63^ and optimized with gapclose v1.12, resulting in 7,228,520 bp of high-quality sequences with 100% coverage of the estimated genome. The integrity and contamination of the genome were assessed using CheckM v1.2.3^64^. The genome was classified as high-quality draft according to MIMAG standards^65^, with 98.5% completeness, 1.2% contamination, and 0.8% strain heterogeneity. The DNA G + C content was directly determined from the genome sequence data. Digital DNA˗DNA hybridization (dDDH) values were determined using the Genome to Genome Distance Calculator (GGDC) v3.0^66^. The average nucleotide identity (ANI) values were calculated using OrthoANI through EzBioCloud, following the algorithm outlined by Yoon et al.^67^. Functional annotation was conducted by comparing sequences with databases such as the Kyoto Encyclopedia of Genes and Genomes (KEGG)^68^, Clusters of Orthologous Groups (COG)^69^, Gene Ontology (GO)^70^, Swiss-Prot^71^, and the Carbohydrate-Active Enzymes (CAZy) database^72^. The draft genome has been deposited in GenBank under the accession number JBHDJG000000000.

### Analysis of phosphate solubilizing activity in solid media

Phosphate solubilization activity was qualitatively assessed using the plate assay on organo-P solid medium (hopebio, HB8549-1) and inorganic P solid medium (hopebio, HB8549-2)^73^. The media supplemented with either 5.00 g/l calcium phosphate or 0.2 g/l lecithin as P source. Strain NGMCC 1.200843^T^ was inoculated at the center of the plates, with *Escherichia coli* L-7 serving as a negative control^74^. The plates were incubated at 37 °C for 7 days. After incubation, the diameters of the clear zone (halo) surrounding the bacterial growth and colony were measured. A clear zone around the colony indicated phosphate solubilization. The phosphate solubilization index was calculated using the formula: Phosphorus solubilization index = (diameter of the halo + diameter of the colony)/diameter of the colony^73,75^. Due to unavailability of reference strains (*Bacillus megamegalorum* ATCC 14581^T^)^76^, activity validation relied on negative controls (*E. coli*) and literature-derived PSI benchmarks.

### Analysis of starch hydrolysis test

The amylase activity of strain NGMCC 1.200843^T^ was assessed using Soluble Starch Medium (hopebio, HB8513-1) supplemented with 1% starch. Strain NGMCC 1.200843^T^ was inoculated into the medium and incubated at 30 °C for 2 days. Formation of a clear zone around the colony indicated complete starch hydrolysis. To further confirm starch hydrolysis, the color reaction was determined after flooding over the 2-week-old culture of strain NGMCC 1.200843^T^ with Lugol’s iodine (hopebio, HB8673-1). Gram iodine reacts with starch to form a dark blue, purple, or black complex depending upon the concentration of iodine.

## Electronic supplementary material

Below is the link to the electronic supplementary material.

Supplementary Material 1

Supplementary Material 2

Supplementary Material 3

## Data Availability

The GenBank accession numbers for 16S rRNA gene sequences of strains NGMCC 1.200843^T^ is PP976594. The draft genome sequences of strains NGMCC 1.200843^T^ has been deposited at NCBI under the accession no. JBHDJG000000000.
