# Supplementary material for: Novel gut bacteria species Paenibacillus ilasis with phosphorus degrading and soluble starch hydrolysis abilities isolated from fresh feces of rhinoceros
Source: Sci Rep. 2025 Jul 1;15:21750. doi: 10.1038/s41598-025-06760-w (PMC12219264; doi:10.1038/s41598-025-06760-w)
Supplement: Supplementary file 1 — Supplementary Material 1 [file 41598_2025_6760_MOESM1_ESM.docx]

**Novel gut bacteria species *Paenibacillus ilasis* with phosphorus degrading and soluble starch hydrolysis abilities isolated from fresh feces of rhinoceros**

Xue Li^1^·Shuyu Zuo^1^·Ming Li^3^·Qin Li^2*^·Lei Su^1*^

*^1^ National Human Diseases Animal Model Resource Center,* *Institute of Laboratory Animal Sciences, Chinese Academy of Medical Sciences (CAMS) & Peking Union Medical College (PUMC), Beijing 100021, China*

*^2^* *College of Biological Sciences, China Agricultural University, Beijing 100193, China*

*^3^ Institute of Animal Science, Chinese Academy of Agricultural Sciences, Technology Support Platform, Beijing 100193, China*

*Correspondence author: Lei Su

*E-mail: sulei@pumc.edu.cn;*

Qin Li

*E-mail:liqin@cau.edu.cn*


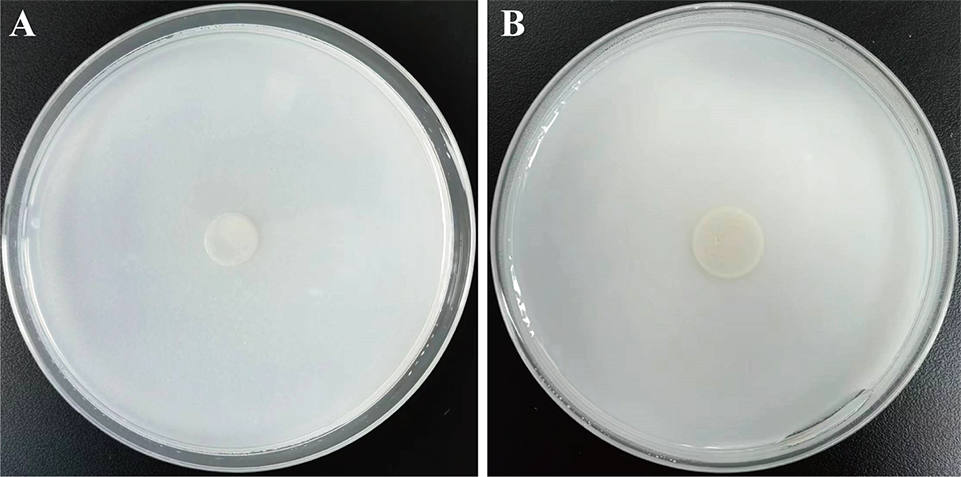


Fig. S1 – Qualitative analysis of inorganic P solubilization ability. A: *Escherichia coli* L-7 as negative control; B: Strain NGMCC 1.200843^T^ cultivated on calcium phosphate as P source medium plates.
