# Supplementary material for: Novel gut bacteria species Paenibacillus ilasis with phosphorus degrading and soluble starch hydrolysis abilities isolated from fresh feces of rhinoceros
Source: Sci Rep. 2025 Jul 1;15:21750. doi: 10.1038/s41598-025-06760-w (PMC12219264; doi:10.1038/s41598-025-06760-w)
Supplement: Supplementary file 2 — Supplementary Material 2 [file 41598_2025_6760_MOESM2_ESM.docx]

**Table S1.** Complete comparative phenotypic, chemotaxonomic, and genomic data of strain NGMCC 1.200843^T^ and closely related type species of the genus *Paenibacillus*. Strains: 1, NGMCC 1.200843^T^; 2, *P. lautus* DSM 3035^T^; 3, *P. glucanolyticus* DSM 5162^T^; 4, *P. qingshengii* JCM 30613^T^; 5, *P. solani* FJAT-22460^T^. Data were obtained in this study unless indicated. +, Positive; ˗, negative; w, weakly positive.

| Characteristics | 1 | 2 | 3 | 4 | 5 |
| --- | --- | --- | --- | --- | --- |
| **API ZYM results** | | | | | |
| Alkaline phosphatase | + | ND | ND | – | + |
| Esterase(C4) | + | ND | ND | + | + |
| Lipid esterase(C8) | + | ND | ND | + | + |
| Lipoidase(C14) | – | – | w | – | – |
| Leucine arylamidase | + | ND | ND | + | + |
| Valine arylamidase | w | ND | ND | – | + |
| Cystine arylamidase | + | ND | ND | – | – |
| Trypsin | w | ND | ND | – | – |
| Chymotrypsin | + | w | – | – | – |
| Acid phosphatases | + | ND | ND | – | – |
| Naphthol–AS˗BI–phosphohydrolase | + | – | – | + | + |
| α–galactosidase | + | ND | ND | – | – |
| β–galactosidase | + | ND | ND | + | + |
| β–glucuronidase | w | ND | ND | – | – |
| α–glucosidase | + | ND | ND | – | – |
| β–glucosidase | + | – | + | – | – |
| N–Acety–β–glucosaminidase | + | ND | ND | – | – |
| α–mannosidase | – | ND | ND | – | – |
| α–fucosidase | – | ND | ND | – | – |
| Catalase | + |  | + | – | + |
| Oxidase | + |  | – | – | + |
| **API 50CH results** | | | | |  |
| Glycerol | + | + | + | + | – |
| Erythritol | + | – | – | + | – |
| D–arabinose | + | – | – | + | – |
| L–arabinose | + | + | + | + | + |
| Ribose | + | + | + | + | + |
| D–xylose | + | + | + | + | + |
| L–xylose | + | – | – | – | – |
| Adonitol | + | – | – | + | – |
| Methyl–β–D–xylopyranoside | + | + | + | – | – |
| Galactose | + | + | + | + | + |
| Glucose | + | + | + | + | + |
| Fructose | + | + | + | + | + |
| Mannose | + | + | + | + | + |
| Sorbose  Rhamnose | + | – | – | + | – |
|  | + | – | – | + | – |
| Dulcitol | + | – | – | + | – |
| Inositol | + | – | – | + | – |
| Mannitol | + | + | + | + | + |
| Sorbitol | + | – | – | + | – |
| Methyl–α–D–mannopyranoside | + | – | + | – | – |
| Methyl–α–D–glucopyranoside | + | + | + | + | w |
| N–acetylglucosamine | + | + | + | + | + |
| Amygdalin | + | + | + | + | w |
| Arbutin | + | + | + | + | + |
| Esculin ferric citrate | + | – | – | + | – |
| Salicin | + | + | + | + | – |
| D–cellobiose | + | + | + | + | – |
| D–maltose | + | + | + | + | – |
| D–lactose | + | + | + | + | + |
| D–melibiose | + | + | + | + | – |
| D–sucrose | + | + | + | + | + |
| D–trehalose | + | + | + | + | + |
| Inulin | + | – | + | – | – |
| D–melezitose | + | – | – | – | – |
| D–raffinose | + | + | + | + | – |
| Starch | + | + | + | + | + |
| Glycogen | + | + | + | + | + |
| Xylitol | + | – | – | + | – |
| Gentiobiose | + | + | + | – | + |
| D–turanose | + | + | + | – | + |
| D–lyxose | + | – | – | – | – |
| L–fucose | + | – | – | – | – |
| D–fucose | + | – | – | – | – |
| D–tagatose | + | – | – | – | – |
| D–arabitol | + | – | – | + | + |
| L–arabitol | + | – | – | – | – |
| Gluconate | + | – | – | – | + |
| 2–keto–D–gluconate | – | – | – | + | w |
| 5–keto–D–gluconate | + | – | – | – | – |
| **API 20E results** | | | | |  |
| ONPG hydrolysis | + | + | – | ND | + |
| Arginine dihydrolase | – | – | – | + | – |
| Lysine | – | – | – | ND | ND |
| Ornithine | – | – | – | ND | – |
| Citrate | – | – | – | – | + |
| Hydrogen sulfide(H_2_S) | – | – | – | ND | – |
| Urease | – | – | – | ND | + |
| Tryptophan | – | – | – | ND | – |
| Indole | – | – | – | – | + |
| Pyruvate | – | ND | ND | ND |  |
| Gelatin | – | – | – | + | – |
| Glucose | + | + | + | + | + |
| Mannose | + | + | + | + | + |
| Inositol | – | – | – | + | – |
| Sorbitol | – | – | – | + | – |
| Rhamnose | + | – | – | + | – |
| Sucrose | + | + | + | + | + |
| Melibiose | + | + | + | + | – |
| Amygdalin | + | + | + | + | w |
| Arabinose | + | – | + | + | ND |
| Distinctive major fatty acid | C_16:0_,  Iso-C_16:0_,  anteiso-C_15:0_ | C_16:0_,iso-C_16:0_, anteiso-C_15:0_, anteiso-C_17:0_ | Anteiso-C_15:0_ | Iso-C_16:0_,  Anteiso-C_15:0_, | C_16:0_, iso-C_16:0_,  anteiso-C_15:0_ |
| Major polar lipids | PG,PE,DPG,PC,  PL1-2 | DPG,PG,PE,PL1-2,APL1-2,L1 | DPG,PG,PE,  PL1-2,APL,L1-2 | DPG,PG,PE,  PL1-2, L1-2 | PG,PE,DPG,  PL1-2,APL1-2 |
| DNA G+C content(mol%) | 49.9 | 48.4 | 25.7 | 49.9 | 50.9 |
| Optimum pH | 7.0 | 7.0 | 7.0 | 7.0 | 7.0 |
| pH range | 6.0–8.0 | 6.0–10.8 | 6.5–11.0 | 6.0–11.0 | 5.0–12.0 |
| Temperature range(°C) | 20–37 | 4–50 | 15–37 | 10–37 | 10–40 |
| Optimum temperature(°C) | 30 | 30 | 30 | 37 | 30 |
| NaCl range(%) | 0–2.0 | 0–10.0 | 0–9.0 | 0–3.0 | 0–5.0 |
| Optimum NaCl(%) | 1.0 | ND | 0 | 1.0 | 0 |
| Motility | + | ND | + | + | + |
| Endospore formation | + | ND | – | + | + |
| Cell shape | Rod | Rod | Rod | Rod | Rod |
| Cell length(μm) | 2.5–4.0 | 1.6–8.0 | 1.0–1.2 | 4.0 | 1.6–3.3 |
| Cell width(μm) | 0.4–0.5 | 0.8–1.3 | 0.6–0.7 | 1.0 | 0.4–0.7 |
| Gram staining | negative | positive | positive | negative | positive |
